# The Midwives of Greece, and Their Remedies

**Published:** 1844-06-01

**Authors:** X. Landerer

**Affiliations:** Professor of the University of Athens


### THE MIDWIVES OF GREECE, AND THEIR REMEDIES.

By X. Landerer, M. D., Professor of the University of Athens.

The ignorance and superstition of the Greek midwives are very injurious to their patients. Their remedies are curious, kept secret, and are employed without consulting the physician. They consist either in such as promote menstruation, or in such as remove sterility. To promote menstruation, they use strong emmenagogues, as aloes, crocus, and rubia tinctorum, and they consider fresh resin of firs, prepared with turpentine, an infallible nostrum in these cases, and administer it, made up with bread into a sort of bolus, weighing about one drachm. Should this dose not have the desired effect, they administer a second and a third, and recommend draughts of camomile tea, or decoction of matricaria parthenium, or of herba aurantiorum, to be taken afterwards. They also use, for the same end, vapour baths in the following way:—The patient is placed on a close stool, filled with boiling water, in which rue and orange leaves are digesting. After she has been exposed for some time to the vapours, she is brought to bed, and receives some cups of rue tea. This last mode is said to fail very rarely. Their treatment of *sterility* is more dangerous. As a general rule, the barren female seeks first the assistance of midwives, who generally employ violent and exceedingly stimulating remedies, introduced at once, *per vaginam.* Among others, they use boluses of myrrh, camphor, oil of camomile, pinks, and cinnamon, cotton *tampons,* with tincture of castoreum, moschus, and even cantharides! Onions, garlic, vapour baths, with herbæ rutæ, and fresh orange leaves, are also used. This unwarrantable treatment is often, of course, followed by dangerous local inflammations. In difficult and protracted labours, they often administer brandy or rum, with pepper and cinnamon, in such doses as to intoxicate the patient. The oil of almonds is also much used. *Some midwives are in the habit of shaking the patient very violently, in order to promote delivery.* The new-born child is nursed in the usual way, with the exception that the midwives consider dangerous—cleanliness, frequent washing, and combing. All diseases of infants are, according to midwives, caused by colic, and *syrupus rhei* is given as a specific. If the child becomes very restless, they give it poppy-juice, which they prepare themselves from the papaver rhœas. If the child remains still restless, they give it a panada prepared from wine and biscuit. The children, of course, become intoxicated, and often lie for days and nights in a soporific state. The abuse of these remedies, and the excessive swathing of the children, are, no doubt, the causes of so many of them dying by hydrocephalus. If the child cannot be pacified by opium, nor by its vinous food, it is supposed to suffer from flatulence. To remove this, the midwives perform an exceedingly cruel operation; namely, the inversion of the navel. They bore a finger into the navel of the child, and turn it round several times. The cries of the children show the incredible pains caused by this operation, which, however, generally removes the flatulence. Umbilical ruptures are thus often caused. We know, that in consequence of determination of blood towards the abdomen, the movements of the living fœtus are sometimes not felt by the mother. In such cases, the Greek female immediately applies to the midwife, who roasts a few pieces of cheese, which she lays on the belly in the form of a cross, and makes the patient eat some of it. Should the child not move now, a second remedy is employed, which consists in boiling dates, and binding them on the belly. Should both these remedies fail the life of the child is despaired of. The midwife, resorting to her last resource, takes a mouthful of rum or brandy, and spirts it with force in a fine stream through the teeth on the naked belly of the pregnant woman, whose eyes are generally blindfolded to prevent her from witnessing the operation. This remedy, I have been assured, according to the assertions of many women, is infallible, and promptly elicits the child's movements, if it be alive. To *promote the secretion of milk* in the suckling woman, the Greek midwives chiefly use the *milk of hazel-nuts.* The hazel-nut kernels are made into an emulsion with melon-seeds and sugar. *In three cases* I witnessed *a considerable increase in the secretion of milk* after the use of some glasses of this emulsion.—*Medical Times,* April 13, 1844.

